# Human soluble CD39 displays substrate inhibition in a substrate-specific manner

**DOI:** 10.1038/s41598-023-36257-3

**Published:** 2023-06-02

**Authors:** Venkat M. K. Vadlamani, Kavinda K. J. Gunasinghe, Xavier W. Chee, Taufiq Rahman, Matthew T. Harper

**Affiliations:** 1 Department of Pharmacology, University of Cambridge, Tennis Court Road, Cambridge, CB2 1PD UK; 2 Swinburne University of Technology Sarawak, Kuching, Malaysia

**Keywords:** Extracellular signalling molecules, Enzyme mechanisms

## Abstract

CD39 (ectonucleoside triphosphate diphosphohydrolase-1; ENTPD1) metabolizes extracellular ATP and ADP to AMP. AMP is subsequently metabolized by CD79 to adenosine. CD39 activity is therefore a key regulator of purinergic signalling in cancer, thrombosis, and autoimmune diseases. In this study we demonstrate that soluble, recombinant CD39 shows substrate inhibition with ADP or ATP as the substrate. Although CD39 activity initially increased with increasing substrate concentration, at high concentrations of ATP or ADP, CD39 activity was markedly reduced. Although the reaction product, AMP, inhibits CD39 activity, insufficient AMP was generated under our conditions to account for the substrate inhibition seen. In contrast, inhibition was not seen with UDP or UTP as substrates. 2-methylthio-ADP also showed no substrate inhibition, indicating the nucleotide base is an important determinant of substrate inhibition. Molecular dynamics simulations revealed that ADP can undergo conformational rearrangements within the CD39 active site that were not seen with UDP or 2-methylthio-ADP. Appreciating the existence of substrate inhibition of CD39 will help the interpretation of studies of CD39 activity, including investigations into drugs that modulate CD39 activity.

## Introduction

CD39 (ectonucleoside triphosphate diphosphohydrolase-1; ENTPD1) is a critical regulator of purinergic signalling. CD39, an ectonucleotidase, metabolises its substrates ATP and ADP in the extracellular milieu to AMP. AMP is subsequently degraded to adenosine by CD73, an ectonucleotidase commonly co-expressed with CD39^1,2^. Both CD39 and CD73 are essential regulators of purinergic signalling, limiting the extracellular concentration of ATP and ADP and working in conjunction to enhance adenosine signalling^2^. CD39 also metabolises extracellular UTP and UDP to UMP, limiting the extracellular concentration of these purinergic agonists^3^. CD39 activity is enhanced in tumour cells, and through the resulting increased adenosine signalling, CD39 inhibits the immune system^4^. In contrast, in autoimmune conditions, CD39 expression is defective, and through the consequent reduced adenosine signalling, immune system activation is enhanced^5^. Furthermore, CD39 activity regulates blood platelet activation by reducing the availability of platelet activators ATP and ADP and increasing adenosine signalling, which inhibits platelet activation. In thrombosis, CD39 expression is inversely associated with platelet activity, and the soluble form CD39 has been proposed as a potential therapy for platelet-mediated thrombotic diseases^6^. Therefore, understanding the regulation of CD39 activity is important for understanding the contribution of purinergic signalling to cancer, autoimmune conditions, and cardiovascular disease.

Substrate inhibition is a phenomenon in which, in the presence of excess substrate, the activity of the enzyme reaches a maximum and then decreases to a non-zero asymptote or a zero asymptote^7,8^. The phenomenon is widespread and is thought to affect nearly 25% of all known enzymes^9^. The precise mechanism of substrate inhibition is unknown. However, one plausible mechanism is that, in the presence of excess substrate, two or more substrate molecules bind simultaneously to the active site. This unproductive binding prevents the conversion of the substrate into products, thereby inhibiting the enzyme^9^. Substrate inhibition is distinct from product inhibition, in which the reaction product binds to an allosteric site on the enzyme, inhibiting its activity. Since substrate inhibition is a phenomenon that negatively modulates the activity of the enzyme, it is important to investigate if CD39 demonstrates substrate inhibition.

In this study we demonstrate that soluble, recombinant CD39 shows substrate inhibition with ADP or ATP as the substrate. Although the reaction product, AMP, inhibits CD39 activity, insufficient AMP is generated under our conditions to account for the inhibition seen. Comparison with other di- and triphosphorylated nucleosides indicated that substrate inhibition with ADP as a substrate was particularly pronounced compared to other diphosphorylated substrates. However, addition of a methylthio- group to ADP substantially reduced substrate inhibition. Using molecular dynamics simulations we demonstrate that the chemical structure of the base of the substrate substantially alters the stability of substrate interaction with CD39. Appreciating the existence of substrate inhibition of CD39 activity will help the interpretation of studies of CD39 activity, including investigations into molecules that modulate CD39 activity.

## Results

### Soluble CD39 displays substrate inhibition with adenine nucleotides

**Figure 1:**
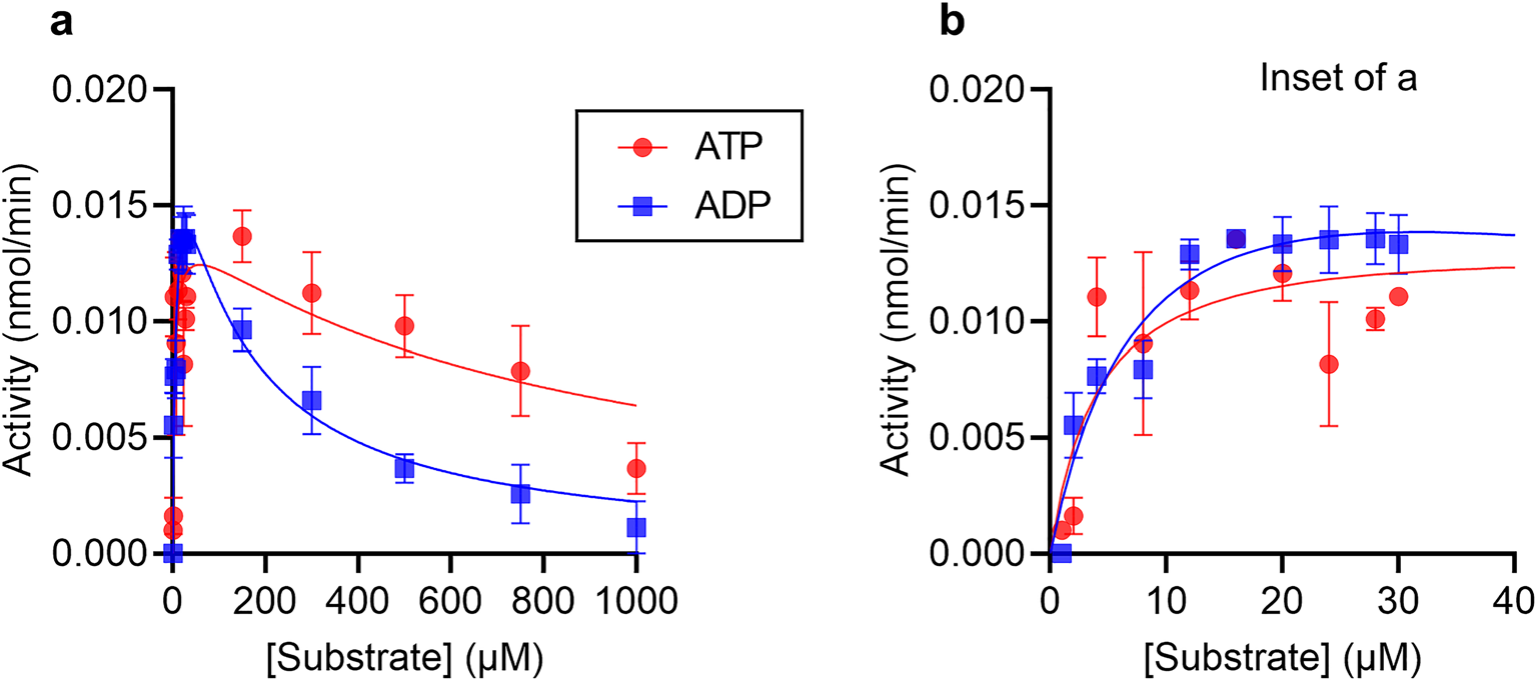
CD39 activity shows substrate inhibition. (**a**) CD39 activity was measured in the presence of varying concentrations of substrate, either ATP or ADP. Data are mean ± s.e.m. (n = 3) and are fitted to a substrate inhibition model as described in the “Methods”. (**b**) Shows the same data for the lowest substrate concentrations, for clarity.

To monitor the activity of CD39, recombinant enzyme was incubated with varying concentrations of substrates, ADP or ATP, and released phosphate measured. (Structures of substrates are shown in Supplementary Fig. 1). As expected, reaction rate increased with increasing substrate concentration (Fig. 1). However, as substrate concentration increased further, reaction rate decreased. Although this trend does not fit standard Michael-Menten kinetics, the data fitted a substrate inhibition model (Fig. 1). The fitted *V*_*max*_, *K*_*M*_ and *K*_*i*_ values from this model are given in Table 1. To the best of our knowledge, substrate inhibition has not been previously reported for soluble human CD39.

**Table 1:** Estimated enzyme kinetic parameters for each substrate.

Substrate	*V*_*max*_ (nmol/min)	*k*_*cat*_ (s^-1^)	*K*_*M*_ (μM)	*K*_*i*_ (μM)
ADP	0.021	17.8	5.71	358
ATP	0.020	17.0	4.01	818
2-MeS-ADP	0.021	17.8	9.36	16,342
2-MeS-ATP	0.018	15.3	5.37	1815
UDP	0.025	21.2	13.27	7538
UTP	0.024	20.4	9.38	1958
GDP	0.022	18.7	26.58	2992
GTP	0.021	17.8	20.61	680

The data were fitted to the Haldane substrate inhibition model in Prism v.9 (Graphpad). The curves are shown in Figs. 1, 3 and 4.

### AMP inhibits CD39 activity

**Figure 2:**
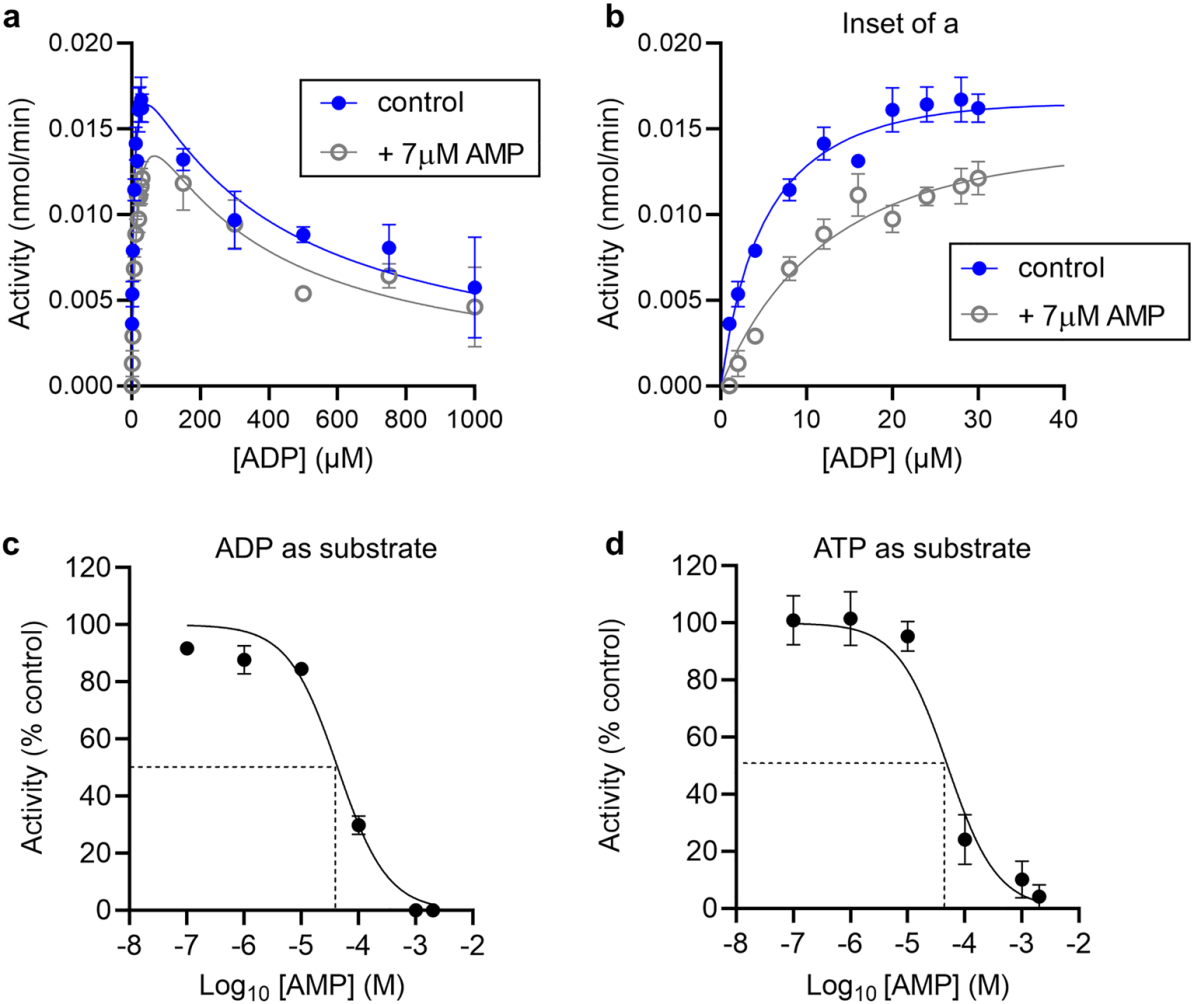
AMP inhibits CD39. (**a**) CD39 activity was measured at varying concentrations of substrate (ADP) in the presence or absence of AMP (7 μM). Data are mean ± s.e.m. (n = 3) and are fitted to a substrate inhibition model as described in the “Methods”. (**b**) Shows the same data for the lowest substrate concentrations, for clarity. (**c**, **d**) CD39 activity was measured in the presence of varying concentrations of AMP, with 24 μM substrate (ADP in **c**, ATP in **d**). Data are normalised to CD39 activity in the absence of AMP (control = 100%). n = 3.

An alternative explanation of the data in Fig. 1 is that AMP, the product of the reaction, can inhibit CD39 activity, either by competing with the substrate or by binding to an allosteric regulatory site. As the substrate concentration is increased, the concentration of AMP formed would increase, and so potentially giving greater inhibition of CD39 activity. The effect of AMP on CD39 activity was therefore assessed. The maximum AMP concentration formed in the assay was approximately 7.2 μM [0.012 nmol/min × 30 min ÷ 50 µl sample]. We therefore examined the effect of 7 μM AMP on CD39 activity (Fig. 2A, B). This concentration of AMP had a small inhibitory effect (most clearly seen in Fig. 2B). However, since this inhibition is much smaller that the extent of inhibition seen at high substrate (ADP) concentration (Fig. 2A), the AMP accumulating in the assay cannot account for the inhibition seen at higher substrate concentration. A much higher concentration of AMP is required for extensive inhibition of CD39. The IC_50_ of AMP was approximately 43 μM with ADP as substrate and 50 µM with ATP as substrate (24 μM substrate in both; Fig. 2C, D). Together, these data suggest that the decrease in CD39 activity at high concentrations of substrate represents substrate inhibition and not product inhibition by AMP.

### Substrate inhibition of CD39 occurs in a substrate-specific manner

**Figure 3:**
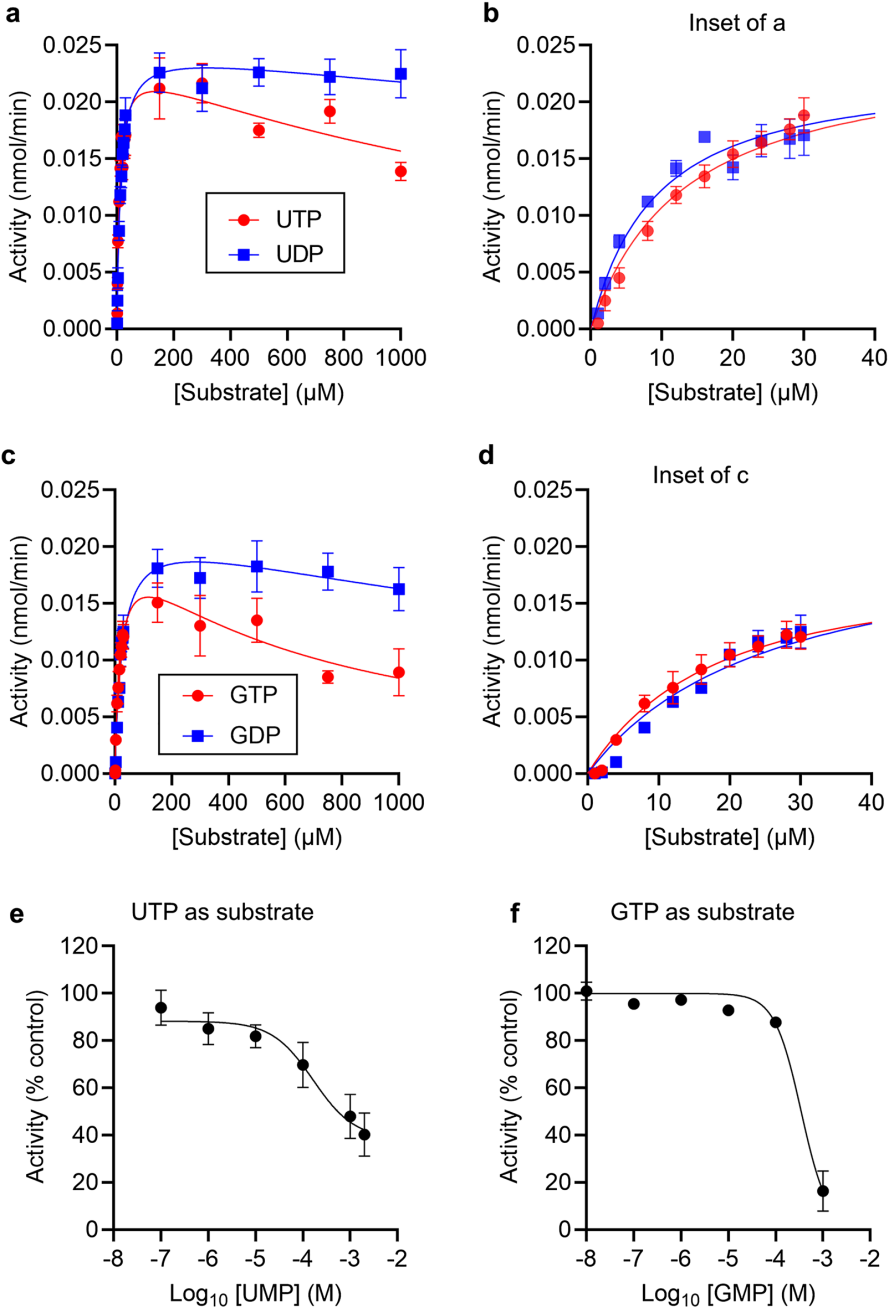
CD39 activity shows substrate inhibition with UTP and GTP. (**a, c**) CD39 activity was measured in the presence of varying concentrations of substrate, either UTP or UDP (**a**), or GTP or GDP (**c**). Data are mean ± s.e.m. (n = 3) and are fitted to a substrate inhibition model as described in the “Methods”. (**b, d**) show the same data for the lowest substrate concentrations, for clarity. (**e**, **f**) CD39 activity was measured in the presence of varying concentrations of UMP (**e**) or GMP (**f**), with 24 μM substrate (UTP in **e**, GTP in **f**). Data are normalised to CD39 activity in the absence of UMP or GMP (control = 100%). n = 3.

UDP and UTP are also metabolised by CD39^3^. We therefore investigated whether substrate inhibition occurs when UDP and UTP are the substrates. As shown in Fig. 3A, weak substrate inhibition was seen with high concentrations of UTP, and almost no substrate inhibition seen with high concentrations of UDP. As expected, at low substrate concentrations, reaction rate increased with increasing substrate concentration (Fig. 3B). The parameters from fitting the substrate inhibition model are given in Table 1. These data indicate that the presence of substrate inhibition depends on the substrate itself. To further investigate this effect, GDP and GTP were used as substrates (Fig. 3C, D). Although CD39 released phosphate from GDP and GTP, the *K*_*M*_ was higher than for the adenine- or uridine-based nucleotides. Substrate inhibition was seen with high concentrations of GTP but not at high concentrations of GDP (*K*_*i*_ > 1 mM; Table 1; Fig. 3C, D). The products of these reactions, UMP and GMP, inhibited CD39 activity with low potency (IC_50_ approximately 165 μM for UMP and 350 μM for GMP; Fig. 3E, F).

**Figure 4:**
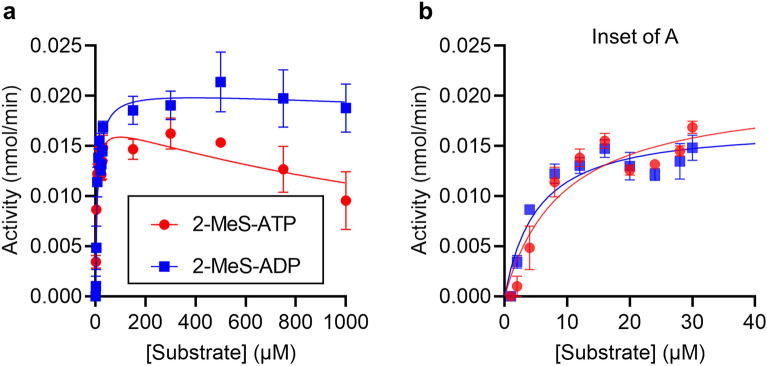
CD39 activity with 2-MeS-ATP or 2-MeS-ADP as substrate. (**a**) CD39 activity was measured in the presence of varying concentrations of substrate, either 2-MeS-ATP or 2-MeS-ADP. Data are mean ± s.e.m. (n = 3) and are fitted to a substrate inhibition model as described in the “Methods”. (**b**) Shows the same data for the lowest substrate concentrations, for clarity.

The data with different substrates indicated that changing the base of the nucleotide affected whether we observed substrate inhibition. Of the substrates tested so far, ADP showed the greatest substrate inhibition. We therefore investigated the consequence of a small change to ADP, by using 2-methylthio (2-MeS)-ADP and 2-MeS-ATP as substrates. As shown in Fig. 4 and Table 1, 2-MeS-ADP and 2-MeS-ATP were efficiently metabolised by CD39. Surprisingly, 2-MeS-ADP showed no substrate inhibition at high concentrations, indicating that addition of the methlythio-group prevented the substrate inhibition seen with the parent substrate ADP.

### Molecular dynamics simulations of CD39 reveal an additional binding site of ADP within CD39

Molecular dynamics (MD) simulations were used to investigate the different behaviour of CD39 substrates. For this analysis we chose: ADP, which showed the greatest extent of substrate inhibition; UDP, which showed the least extent of substrate inhibition of the natural substrates; and 2-MeS-ADP, for comparison with the parent substrate, ADP. By comparing only dephosphorylated substrates we are able to compare the effect of changes to the base. We treated the substrates as non-metabolised ligands to investigate their early interaction with the enzyme rather than how these interactions might change during substrate hydrolysis. The structure of the extracellular domain of CD39 was modelled in AlphaFold 2.0 implemented in ColabFold^10^. Substrates were blind docked using AutoDock Vina and the best predicted complex for each substrate subjected to 1 μs-long simulations in triplicate. The Root Mean Square Deviation (RMSD) for the protein in each of the replicates for all the complexes is shown in Supplementary Fig. 2. These RMSD plots showed that all proteins had reached equilibrium and did not undergo significant structural changes throughout the simulation durations. For most complexes, a stable equilibrium was achieved within 0.3 μs. The Radius of Gyration (RGyr) of the protein for all simulations of the complexes, which relates to changes in compactness and volume^11^, is shown in Supplementary Fig. 3. In all cases the complexes maintained stable volumes and did not show significant changes in its compactness.

**Figure 5:**
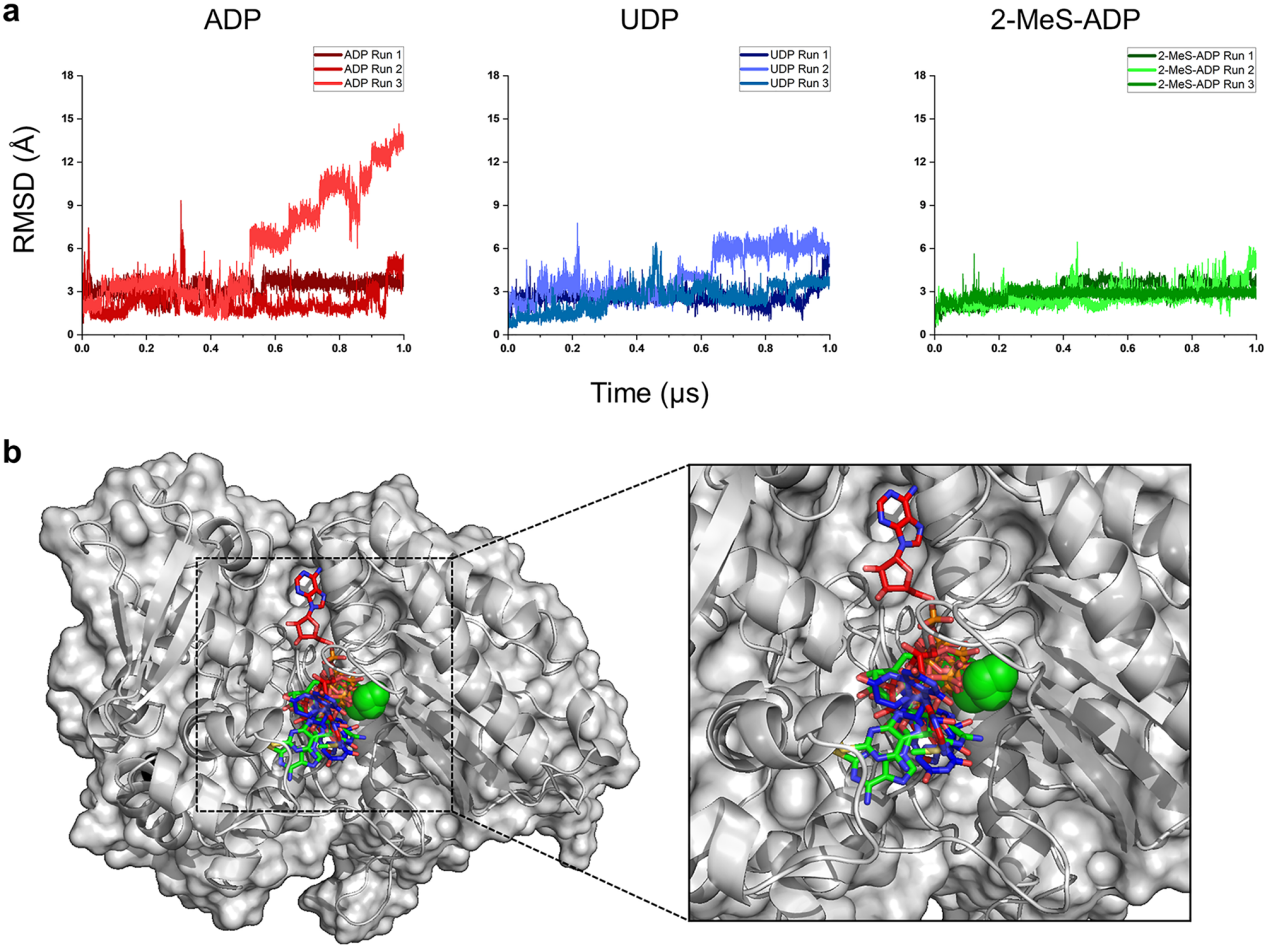
Molecular dynamic (MD) simulation of substrates in CD39 active site. Substrate-bound CD39 was simulated for 1 μs. Root mean square deviation (RMSD) of the substrate is shown in (**a**) for ADP, UDP or 2-MeS-ADP. RMSD are given relative to the first frame of each simulation. (**b**) The final poses of all the ligands. Except ADP in the third replicate all other ligands are within the catalytic site of CD39. ADP, UDP and 2-MeS-ADP are shown in red, blue and green, respectively. The protein surface and the Ca2+ ions are shown in grey and green spheres.

Next, we calculated the RMSD for the ligands for ADP, UDP and 2-MeS-ADP to observe their behaviour^12^. In the case of ADP, the first and second replicates maintained a stable RMSD of 3.61 Å and 2.30 Å, respectively (Fig. 5a). This suggested that the conformations of ADP in the first and second replicates remained stable in the simulations. Visual inspection of the ADP conformation for first and second replicate also showed similar binding pose. However, interestingly, we noted that the ADP in the third replicate started exhibiting large fluctuation in its RMSD at the 0.5 µs-mark (Fig. 5a) with the RMSD increasing upwards until 1 µs without stabilizing. To pursue this further, we extended that simulation by an additional 0.5 µs. ADP in the third replicate reached stability in a different binding conformation at around the 1.10 µs-mark (Supplementary Fig. 4). In contrast, the UDP binding conformation attained stability in all three replicates (RMSD of 2.62 Å, 6.06 Å and 3.02 Å in replicates 1, 2 and 3, respectively) Similarly, 2-MeS-ADP conformations across all three runs also remained stable (RMSD of 3.61 Å, 3.11 Å and 2.88 Å). Visual inspection of the last frame (Fig. 5b) showed that all conformations of ADP, 2-MeS-ADP and UDP (except the conformation of ADP in replicate 3) converged broadly onto the same binding pose with small differences in the positioning of their nucleoside bases.

**Figure 6:**
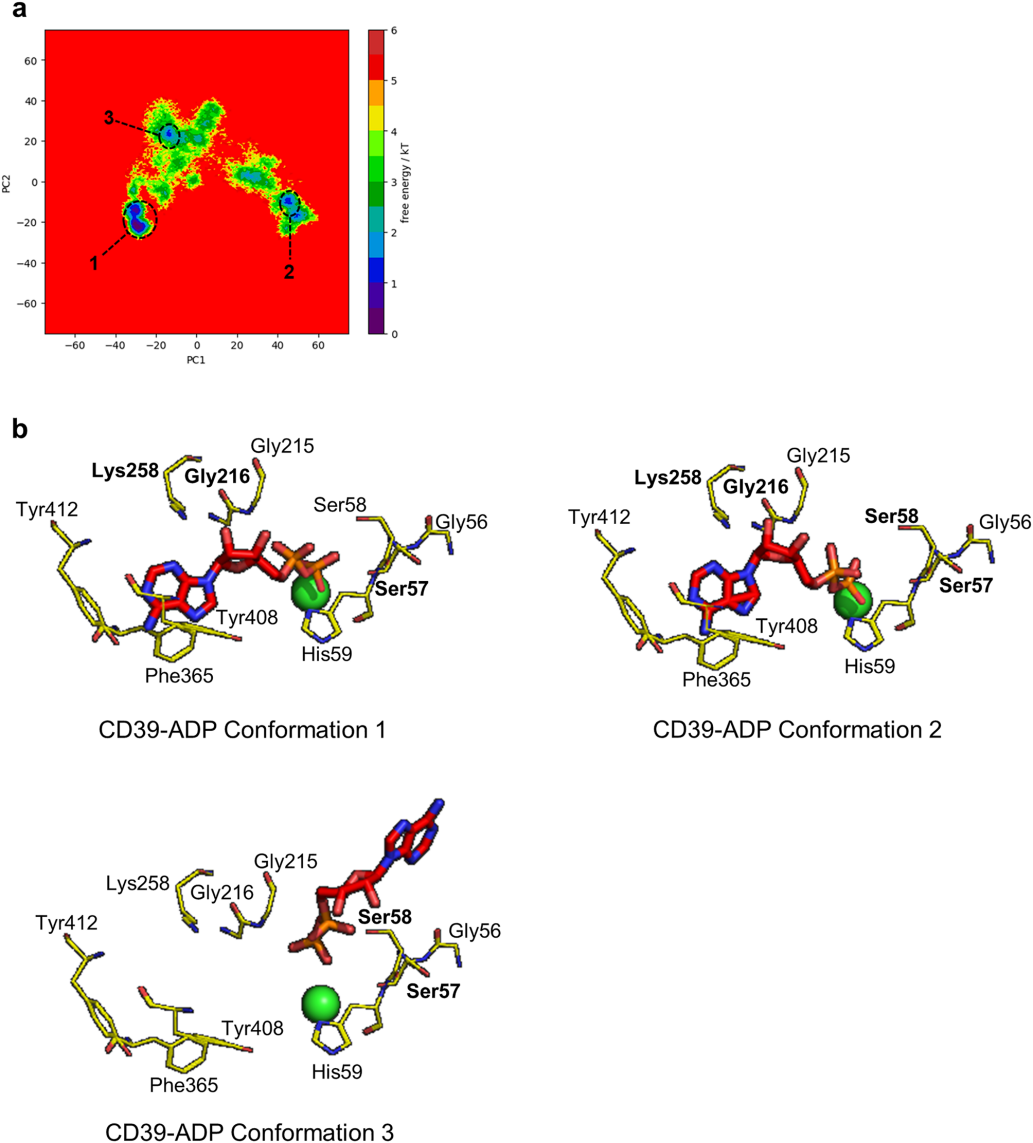
Combined free energy landscape (FEL) of CD39-ADP and most stable conformations. (**a**) The combined FEL plot of CD39-ADP complexes. (**b**) The conformations of the basins in the FEL. The Ca^2+^ ion is shown as a green sphere while the residues that form H-bonds are shown in bold.

**Figure 7:**
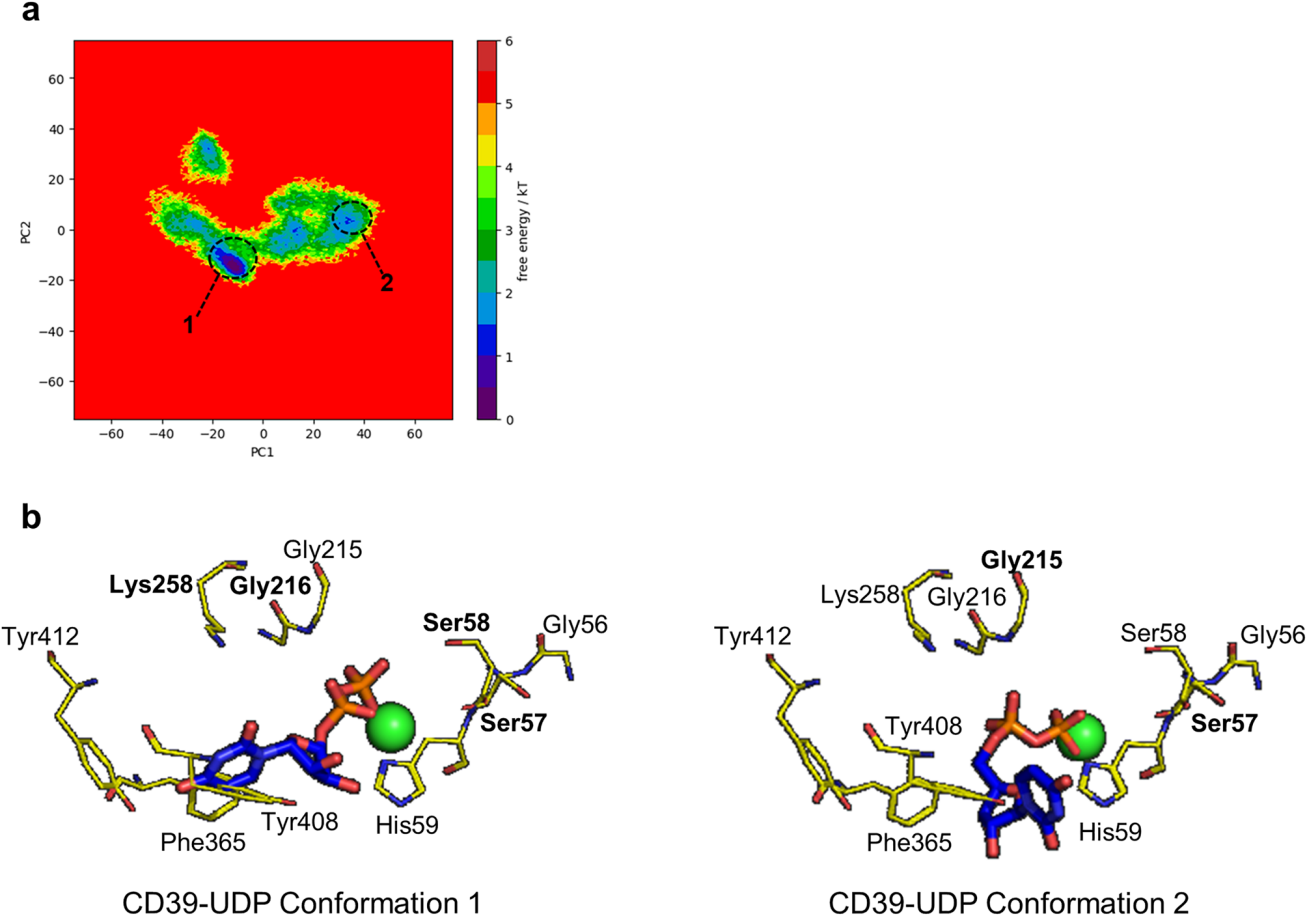
Combined free energy landscape (FEL) of CD39-UDP and most stable conformations. (**a**) The combined FEL plot of CD39-UDP complexes. (**b**) The conformations of the basins in the FEL. The Ca^2+^ ion is shown as a green sphere while the residues that form H-bonds are shown in bold.

**Figure 8:**
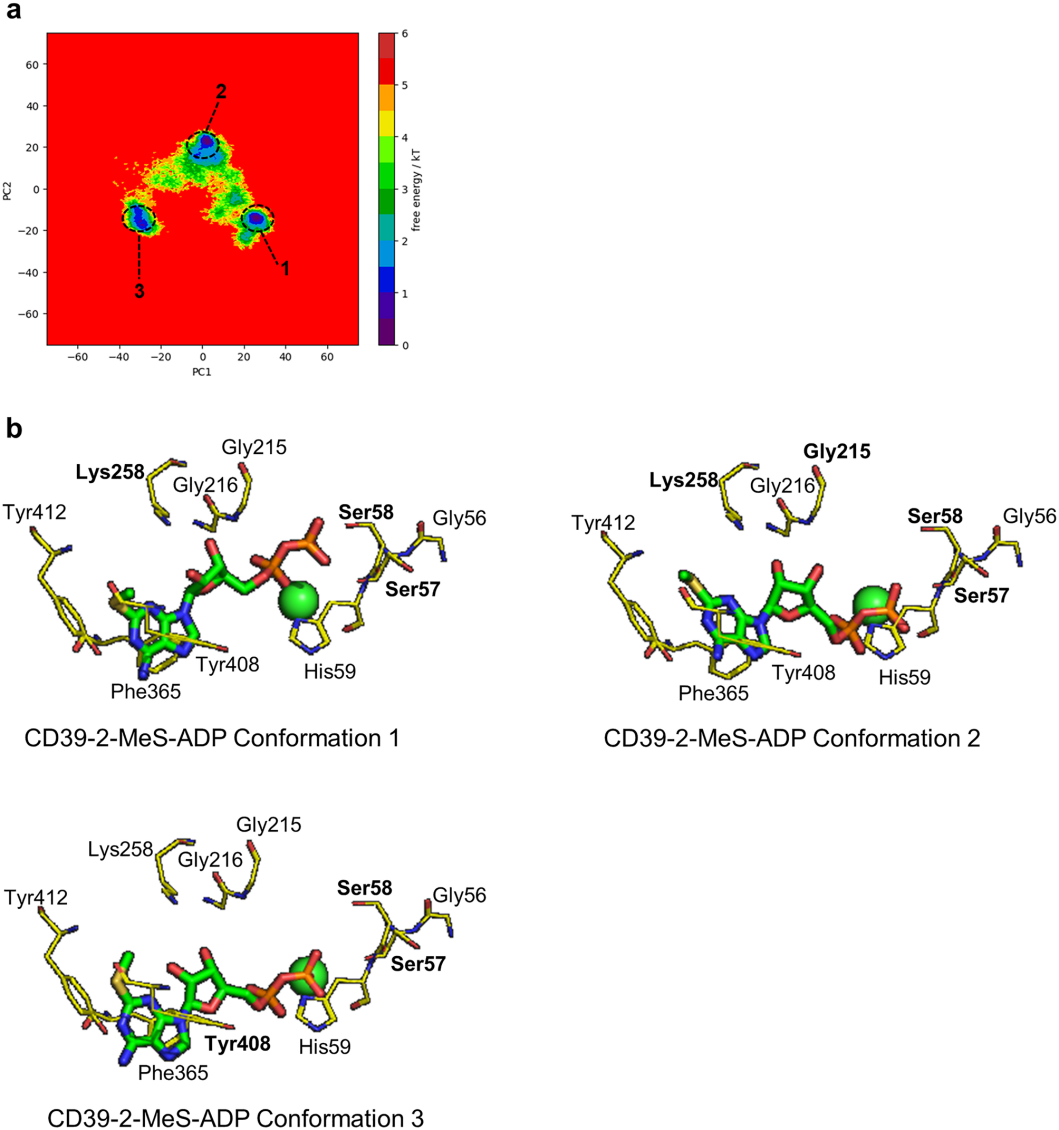
Combined free energy landscape (FEL) of CD39-2-MeS-ADP and most stable conformations. (**a**) The combined FEL plot of CD39-2-MeS-ADP complexes. (**b**) The conformations of the basins in the FEL. The Ca2+ ion is shown as a green sphere while the residues that form H-bonds are shown in bold.

Finally, we studied the Free-energy landscapes (FEL) of the complexes by combining all three replicates using their 1 µs simulations (Figs. 6, 7 and 8). In the combined FEL plot, three basins were seen for both CD39-ADP and CD39-2-MeS-ADP complexes while two basins were seen for CD39-UDP complex. The third basin in ADP achieves this conformation once ADP reached the alternative binding pose (Fig. 6b). In the case of CD39-UDP complex and the CD39-2-MeS-ADP complex, the stable conformations are achieved while the ligand is bound to the catalytic site of CD39 (Figs. 7b, 8b). These simulations reveal that although all substrates readily interact with the activate site of CD39, ADP can occupy an alternative binding site.

## Discussion

CD39 is a critical regulator of purinergic signalling. It degrades ATP and ADP, proinflammatory and prothrombotic mediators. Conversely, together with CD73, it increases the concentration of adenosine, which is broadly anti-inflammatory and anti-thrombotic. CD39 activity therefore impacts autoimmune disorders, immune responses to cancer and arterial thrombosis^4,5,13^.

In standard Michaelis–Menten kinetics, the rate of an enzyme-catalysed reaction rises with increasing substrate concentration, saturating at higher substrate concentrations to reach a stable maximum rate (*V*_*max*_). In our assay, CD39 initially followed this pattern as substrate concentration increased up to approximately 30 μM. This was observed with either ADP or ATP as substrate. However, as the substrate concentration increased further, the rate of reaction decreased. Again, this occurred with either substrate, although it was more evident with ADP. The data were well fitted by the Haldane equation, which describes competitive substrate inhibition^14^.

An alternative explanation that we considered is that the reaction product, AMP, inhibits CD39 activity. Inhibition by product could be by competition for the substrate binding, i.e., acting as a competitive inhibitor. At higher substrate concentration, more AMP would be formed, increasing the inhibition. Alternatively, the product may bind to an allosteric regulatory site, inhibiting enzyme activity in an uncompetitive or non-competitive manner. We investigated these possibilities by adding AMP at the maximum concentration formed during the assay (based on phosphate released). We saw a weak inhibition of CD39 by AMP, but this inhibition was insufficient to explain the extent of reduction of reaction velocity seen with increasing substrate. Moreover, the biphasic effect of substrate on reaction was still evident even when AMP was present at the maximum level generated during our assay conditions. Indeed, the IC_50_ for inhibition by AMP was much greater than expected to be present in the assay. Together, our data support the conclusion that substrate inhibition, rather than product inhibition, is responsible for the decrease in reaction rate. Product inhibition may become more important in vivo if CD73, which converts AMP to adenosine, is inhibited, as it proposed as an anti-cancer therapy^15^.

In this study we used soluble recombinant enzyme. This enzyme consists of 5 apyrase conserved regions (ACRs) that are crucial to nucleotide binding and enzyme activity^16^. However, cellular CD39 is embedded in the plasma membrane by two transmembrane domains. These transmembrane domains affect the activity of CD39^17,18^, and could even affect its susceptibility to substrate inhibition, or product inhibition by AMP. It will be interesting in future studies to investigate whether substrate inhibition occurs in endogenously expressed CD39. Nonetheless, with recombinant CD39, alone or conjugating to targeting proteins, being proposed as an anti-thrombotic therapeutic^13,19^, appreciation of substrate inhibition in recombinant CD39 remains important.

CD39 hydrolyses other di- and tri-phosphorylated nucleotides in addition to ADP and ATP^3^. Interestingly, we also found weak substrate inhibition when UTP and GTP were substrates. Despite ADP showing more stronger substrate inhibition than ATP, little (if any) substrate inhibition was seen for the other di-phosphorylated substrates. This indicates that substrate inhibition occurs in a substrate-specific manner, dependent on the base of the nucleotide. To investigate this further we used 2-MeS-ADP and 2-MeS-ATP. These nucleotides are commonly used to study P2Y receptor signalling. This relatively small change to the adenine base reduced the strength of substrate inhibition. Moreover, greater substrate inhibition was seen with the tri-phosphorylated form, as with GTP/GDP and UTP/UDP, and in contrast to ATP/ADP. The strength of substrate inhibition did not correlate with the *K*_*M*_ for each substrate, suggesting that substrate inhibition is not directly related to the affinity of the substrate for the enzyme. Instead, it appears that the base of the nucleotide substrate may determine the extent to which a substrate may bind to CD39 in an inhibitory manner.

Substrate inhibition has been mechanistically explained in several ways. In the classical model, additional substrate (S) can bind to an enzyme substrate complex (ES), to form ESS. However, product can only be formed from the ES complex, not from ESS^8^. In an alternative model, substrate may bind to the enzyme in a non-productive conformation, excluding further binding of substrate and formation of a productive ES complex^20^. For the latter mechanism, we would predict that ADP, the substrate that showed the greatest extent of substrate inhibition, would be able to bind to CD39 in more than one conformation. We explored this using MD simulations of CD39 with ADP, UDP or 2-MeS-ADP. Although CD39 itself reached equilibrium within the time of each simulation, ADP behaved different to the other two substrates. Although ADP remained relatively stable in the CD39 active site in two replicates, ADP underwent large fluctuations in its binding pose in the third replicate, before eventually stabilizing in a new binding pose. This behaviour was not seen with either UDP or 2-MeS-ADP in any replicate. Interestingly, it was the adenine base of ADP that showed greatest mobility, whereas the two phosphates remained coordinated near the Ca^2+^ ion. The stable poses are revealed as basins in the free energy landscapes (Figs. 6, 7 and 8). Even in conformations 1 and 2 for CD39-ADP, where ADP is similar positions as those seen for UDP and 2-MeS-ADP, the adenine base is further from Phe365/Tyr408/His59. The different positions of the adenine and uridine bases of these substrates was also seen in MD simulations by Iqbal and Shah^21^, indicating that the base of the nucleotide substrates is an important determinant of their binding behaviour, although our study also highlights the greater mobility of ADP. It is likely that ADP binding to CD39 can adopt at least two conformations. We suggest that one of these, conformation 3, is not productive for ADP hydrolysis. Moreover, this non-productive binding would prevent productive binding of substrate, leading to substrate inhibition. Non-productive binding could also prevent effective substrate binding by altering the tertiary structure of CD39 monomers. Alternatively, non-productive binding could disrupt CD39 dimerization^22^. In contrast to ADP, UDP is much less likely to form alternative binding conformation and shows very little substrate inhibition. Moreover, we propose that the addition of the 2-MeS- group to ADP also reduces substrate inhibition. Small chemical modifications to the nucleotide base may therefore influence whether the nucleotide substrate is more likely to form a productive or non-productive ES complex. Interestingly, 8-butylthio (BuS)-ATP and 8-BuS-ADP are resistant to hydrolysis by CD39 and acts as CD39 inhibitors^23^. The direct binding to CD39 forms an unproductive complex, as the 8-BuS-modified nucleotides were not hydrolysed. This supports our proposal that adenine nucleotides can form productive and non-productive ES complexes with CD39 and that small chemical modifications to the adenine base alter the balance between these two states.

The contribution of substrate inhibition to CD39 activity in vivo will depend on the identity of the substrate, as it is more likely with ADP and ATP rather than UTP and UDP. In addition, the contribution of substrate inhibition will also depend on the extracellular concentration of ADP and ATP. Under normal conditions, ATP concentration in human blood and surrounding healthy tissues is sub-micromolar^24^. However, the extracellular ATP concentration may be much higher in the vicinity of cells releasing ATP compared to the surrounding medium^24,25^. Moreover, extracellular ATP concentration may be increased under pathological circumstances. Plasma membrane-targeted luciferase, for example, have shown that the ATP concentration in the tumour microenvironment can reach 50–200 μM^24^. Similarly, computational models predict that extracellular ADP may reach several hundred microM at the base of a thrombus, where ADP and ATP are released by platelets and act in a paracrine manner to promote platelet activation^26^. Red blood cells also release ATP, especially in hypoxic conditions, where local ATP concentration may reach 160 μM^26^. Cell necrosis also leads to substantial local release of ATP and ADP. Under conditions such as these, local concentrations of ADP and ATP may rise sufficiently high that substrate inhibition reduces the local activation of CD39.

In conclusion, soluble recombinant human CD39 shows substrate inhibition in a substrate-dependent manner. This phenomenon may affect the interpretation of in vitro CD39 assays, including those used to screen for novel CD39 inhibitors, and could be relevant in vivo under pathological conditions that lead to substantial elevations of extracellular ATP or ADP concentrations.

## Methods

### Materials

Reagents were obtained from Sigma Aldrich (Poole, UK) unless otherwise stated. Tris Base was obtained from Fisons Analytical Reagent (Cat# T/3712). ADP was obtained from Thermo Fisher Scientific (Cat# 10143940). Recombinant Human CD39/ENTPD1 Protein, CF (Cat# 4397-EN-010) was obtained from R&D System (Biotechne).

### CD39 activity

The recombinant enzyme was prepared in 80 mM Tris. The final enzyme concentrations used in the experiment was 0.02 µg/ml with 5 mM CaCl_2_. Phosphate Standards were prepared with serial dilutions from 50 to 0.39 µM. CD39 activity was monitored as the amount of phosphate liberated from the substrate in 30 min (room temperature). Released phosphate was measured using the Malachite Green Reagent Assay (R&D System Biotechne; Cat# DY996) was performed as per the manufacturer’s instructions. Absorbance was detected at 620 nm in a FluoStar Omega plate reader (BMG LabTech). Absorbance values were converted phosphate concentration using a standard curve generated from the phosphate standards and linear regression.

### Data analysis

Data of CD39 activity with increasing substrate concentration were fitted to a substrate inhibition model using Prism (v9.2; GraphPad) with the equation:

$${\text{Activity }} = V_{max} *\left[ {\text{S}} \right]/\left( {K_{M} + \left[ {\text{S}} \right]*\left( {{1} + \left[ {\text{S}} \right]/K_{i} } \right)} \right)$$

where *V*_*max*_ is the predicted maximum activity if the substrate did not inhibit activity, *K*_*M*_ is the Michaelis-Menton constant, and *K*_*i*_ is the dissociation constant for the substrate binding in such a way that two substrates can bind to the enzyme^27^. *k*_*cat*_ was determined by dividing *V*_*max*_ by the CD39 concentration.

To determine the concentration of AMP that gives 50% inhibition (IC_50_) in presence of ADP or ATP substrate, the activity was normalised to activity in the absence of AMP, then fitted to the equation:

$${\text{Activity}}\;\left( {{\text{\% control}}} \right) = 100/\left( {1 + 10^{{({\text{X}} - {\text{LogIC}}50)}} } \right)$$

where X is the log_10_ of substrate concentration.

Data are presented as mean ± standard error of mean (s.e.m.).

### CD39 homology model and ligand (substrate) docking

The 3D structure of the extracellular domain of human CD39/ ENTPD1 (Uniprot ID: P49961) was modelled using AlphaFold 2.0 implemented in ColabFold^10^. The model was relaxed using the brief AMBER based MD simulation implemented in ColabFold. All the ligands were downloaded from PubChem and blindly docked to the final model of human CD39 using AutoDock Vina^28^ at an exhaustiveness of 24. The best predicted complex for each ligand was then subjected to molecular dynamics simulation.

### Molecular dynamics (MD) simulations

The MD simulations and binding free energy calculations were performed using the AMBER 20 MD package. The simulations were performed in triplicates for all three complexes up to 1 µs. The third replicate of CD39-ADP complex was extended by an additional 0.5 µs to determine whether it reached stability. The ligand charges were parameterized using the *antechamber* module (*GAFF* forcefield)^29^. Note that the ligands are charged at physiological pH (see Supplementary Fig. 1). The complexes were parameterized using the *ff19SB* forcefield, the excess charges of each complex were balanced using Na^+^ ions and TIP3P-modelled water molecules were used to solvate the complexes in a truncated octahedron period box with a 10 Å buffer distance using the *tleap* module^30^. The Particle Mesh Ewald (PME) method was used to treat long-range electrostatic interactions. The non-bonded cut-off was 10 Å in all cases while a timestep of 2 fs was used for each simulation.

Minimization was carried out in two stages for each complex. The first stage consisted of 500 steps of steepest descent followed by 500 conjugate gradient minimizations. The restraint force and the PME cutoff distance were kept at 300 kcal/mol and 10 Å respectively. The second stage comprised the steepest descent of 1000 steps with a conjugate gradient minimization of 1000 without any restraints with a PME cutoff distance of 10 Å. After minimization, heating was carried out for the complexes over 200 ps where the temperature was increased from 0 to 300 K with the NVT ensemble followed by 5 ns of equilibration with the NPT ensemble. Temperature and pressure were kept at 300 K and 1.0 bar respectively for both equilibration and production stages which were monitored by the Langevin thermostat and the Berendsen thermostat. SHAKE algorithm was activated during both stages of the simulation. Analysis of trajectories were performed using the first frame as reference using the cpptraj module^31^. The free energy landscape was calculated using the *pytraj* module for the three complexes by using 12,500 frames from the total simulation^31^.

### Ethical approval

This study did not involve human participants or protected animals.

### Informed consent

This study did not involve human participants, or any material derived from human participants.

## Supplementary Information

Supplementary Figures.

## Data Availability

Data are available from the corresponding author on reasonable request.
