# Supplementary material for: Human soluble CD39 displays substrate inhibition in a substrate-specific manner
Source: Sci Rep. 2023 Jun 2;13:8958. doi: 10.1038/s41598-023-36257-3 (PMC10238538; doi:10.1038/s41598-023-36257-3)
Supplement: Supplementary file 1 — Supplementary Figures. [file 41598_2023_36257_MOESM1_ESM.pdf]

Supplementary Figure 1

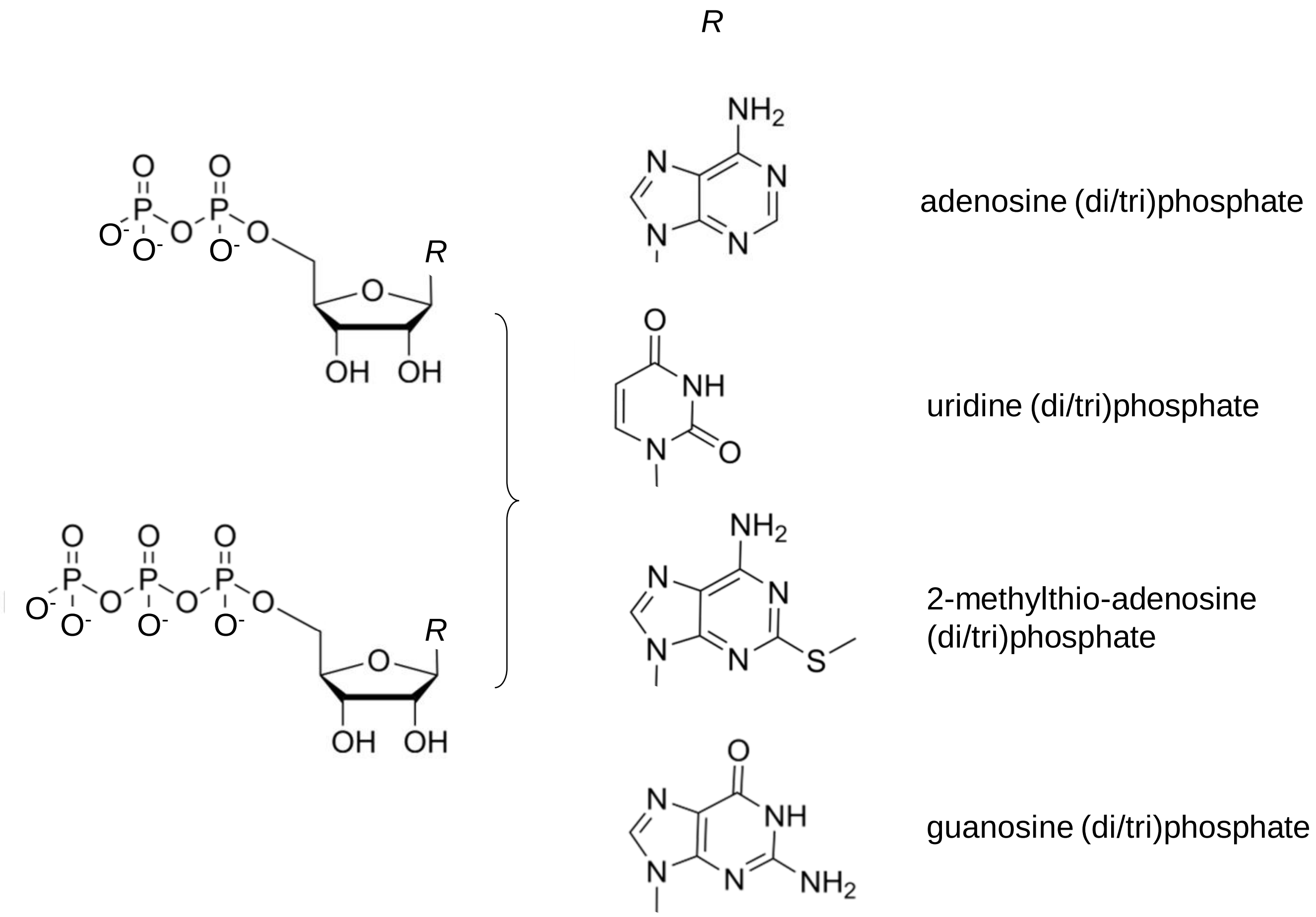

Supplementary Figure 1: Structures of substrates used in the study

Supplementary Figure 2

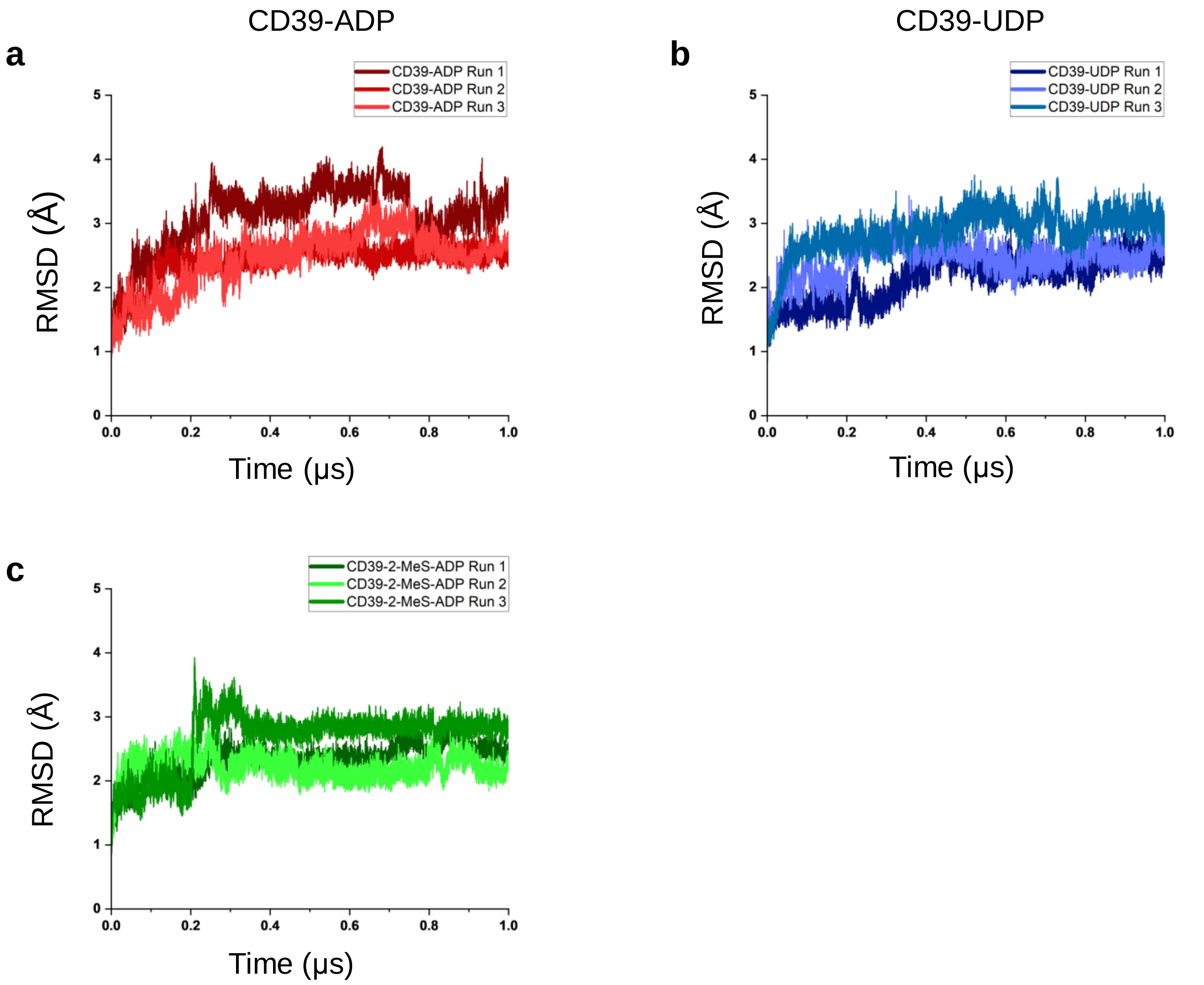

Supplementary Figure 2: The RMSD plots of CD39-substrate complexes. Substrates were blind docked into CD39 then underwent a 1 μs molecular dynamics (MD) simulation. The root mean squared deviation (RMSD) of CD39 is shown for each complex: (**a**) ADP-CD39 complex; (**b**) UDP-CD39 complex; (**c**) 2-MesADP-CD39 complex.

Supplementary Figure 3

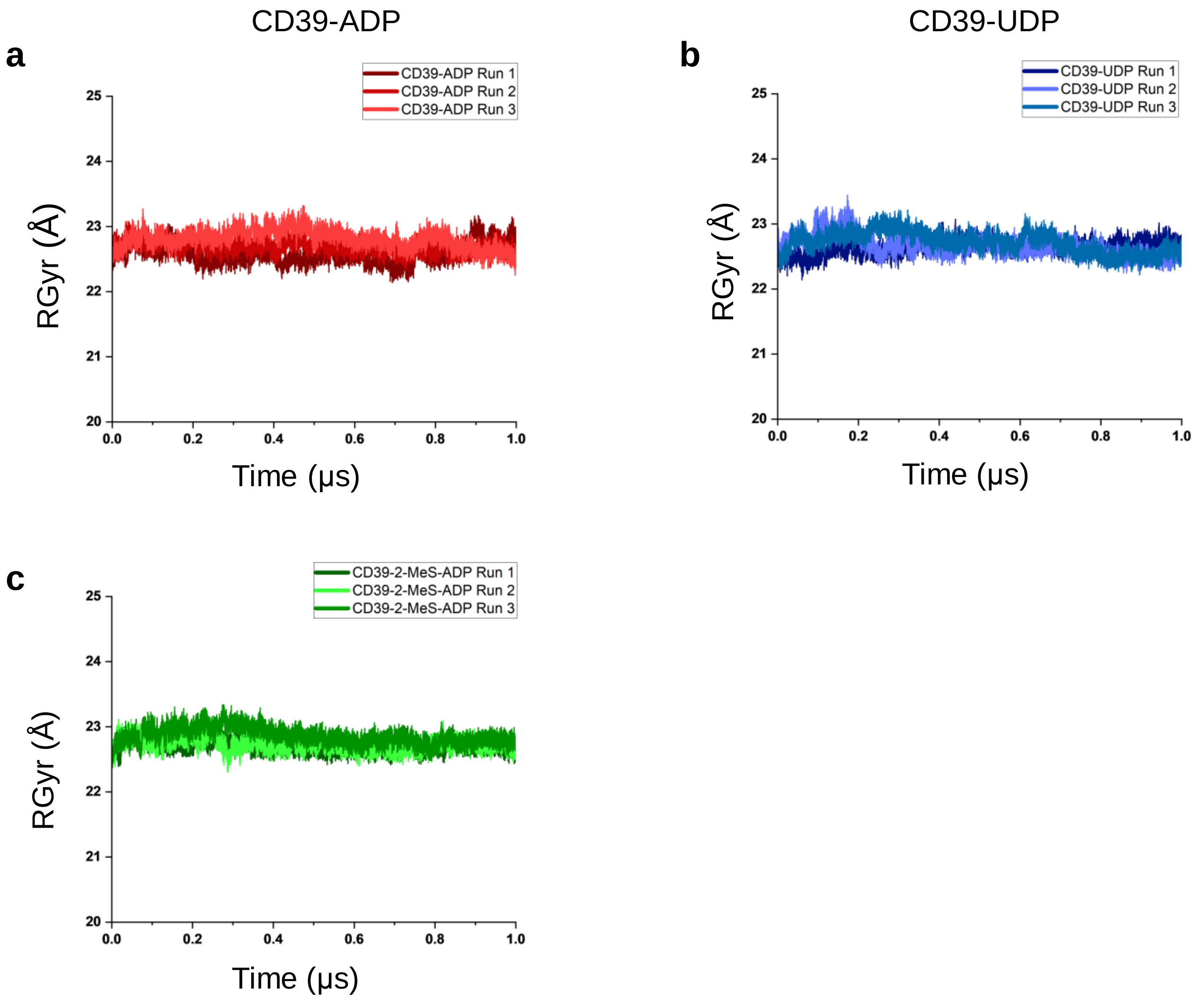

Supplementary Figure 3: The RGyr plots of CD39-substrate complexes. Substrates were blind docked into CD39 then underwent a 1 µs molecular dynamics (MD) simulation. The radius of gyration (RGyr) of CD39 is shown for each complex: (**a**) ADP-CD39 complex; (**b**) UDP-CD39 complex; (**c**) 2-MeS-ADP-CD39 complex.

Supplementary Figure 4

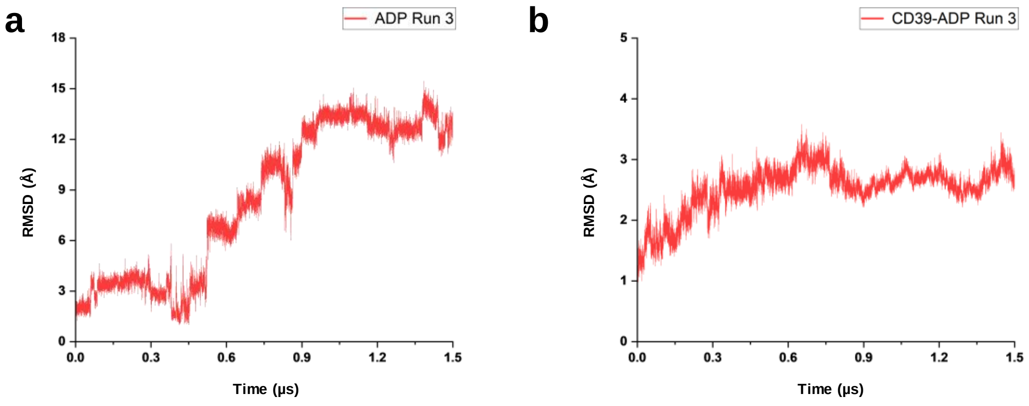

Supplementary Figure 4: RMSD of the extended simulation of the third template of CD39-ADP complex to 1.5 μs. (**a**) The ligand RMSD of ADP in the third simulation. (**b**) The protein RMSD of the third template
